# Insights into the nutritional properties and molecular basis of biosynthesis of amino acids and vitamins of *Gastrodia elata* offered by metabolomic and transcriptomic analysis

**DOI:** 10.3389/fpls.2023.1183139

**Published:** 2023-06-26

**Authors:** Yunsheng Wang, Muhammad Qasim Shahid

**Affiliations:** ^1^ School of Health and Life Science, Kaili University, Kaili City, Guizhou, China; ^2^ State Key Laboratory for Conservation and Utilization of Subtropical Agro-Bioresources, South China Agricultural University, Guangzhou, China; ^3^ Guangdong Provincial Key Laboratory of Plant Molecular Breeding, South China Agricultural University, Guangzhou, China; ^4^ College of Agriculture, South China Agricultural University, Guangzhou, Guangdong, China

**Keywords:** differentially expressed genes, differentially accumulated metabolites, *Gastrodia elata*, metabolome, transcriptome

## Abstract

*Gastrodia elata* Blume (*GE*), a traditional and precious Chinese medicinal material, has been approved as a functional food. However, understanding *GE*’s nutritional properties and its molecular basis remains limited. Here, metabolomic and transcriptomic analyses were performed on young and mature tubers of *G. elata.f.elata* (*GEEy* and *GEEm*) and *G. elata.f.glauca* (*GEGy* and *GEGm*). A total of 345 metabolites were detected, including 76 different amino acids and their derivatives containing all human essential amino acids (e.g., l-(+)-lysine, l-leucine), 13 vitamins (e.g., nicotinamide, thiamine), and 34 alkaloids (e.g., spermine, choline). *GEGm* has higher amino acid accumulation than *GEE*y, *GEEm* and GEGy, and vitamin contents were also slightly different in all four samples. Implying that *GE*, especially *GEGm*, is a kind of excellent complementary food as amino acid nutrition provider. From assembled 21,513 transcripts (genes) based on the transcriptome, we identified many genes that encode enzymes (e.g., pfkA, bglX, tyrAa, lysA, his B, aroA), which are responsible for the biosynthesis of amino acids and enzymes (e.g., nadA, URH1, NAPRT1, punA, rsgA) that related to vitamins metabolism. A total of 16 pairs of the differentially expressed genes (DEG) and differentially accumulated metabolites (DAM) (e.g., gene-tia006709 coding GAPDH and l-(+)-arginine, and gene-tia010180 coding tyrA and l-(+)-arginine) and three DEG-DAM pairs (e.g., gene-tia015379 coding NadA and nicotinate d-ribonucleoside) show significant similar positive or negative correlation based on three, and two comparisons of *GEEy vs. GEGy*, *GEGy vs. GEGm*, GEEy *vs. GEGy* and *GEEm vs. GEGm*, which involved into amino acid biosynthesis, and nicotinate nicotinamide metabolism, respectively. These results prove that the enzyme coded by these DEG promotes (positive correlation) or inhibits (negative correlation) the biosynthesis of parallel DAM in *GE*. Overall, the data and corresponding analysis in this study provide new insights into the nutritional properties of *GE and the* related molecular basis.

## 1 Introduction

*Gastrodia elata* Blume (*GE*) (Orchidaceae) has been used to promote health and treat various neurological disorders such as convulsions, migraines, dizziness, seizures, paralysis, and mood disorders for thousands of years in East Asia (Lin et al., 2018). Modern medical research has found that *GE* has a variety of healthful activities, including anti-depressant (Qiu et al., 2014), antioxidation (Liu and Mori, 1992; Jung et al., 2007), anti-inflammation (Hwang et al., 2009), anti-obesity (Sun et al., 2012) activities, and has been found to improve memory (Hsieh et al., 1997). In addition, *GE* is a high-grade supplementary food in some Chinese dishes and has recently been approved as a functional food by the Chinese government (Shu et al., 2013; Jaswir et al., 2017; Lu et al., 2020; Ma et al., 2021). *GE*’s healthful and nutritional properties are undoubtedly endowed by its unique bioactive compounds that were biosynthesized in tuber tissues. Many studies have been executed to identify the active compounds in *GE* tissues responsible for their beneficial health effects. The first reported biochemical compound of *GE* was vitamin A, identified in 1936 (Liu, 1961). Then, vanilla alcohol was identified in the middle of the last century (Liu and Yang, 1958). Gastrodin (p-hydroxymethylphenyl-β-d-glucopyranoside), *GE*’s most important medicinal compound, was first identified in 1979 (Feng et al., 1979). In addition, at least sixteen gastrodin derivatives were identified in the tubers of *GE* (Xu et al., 2019). So far, approximately 100 bioactive compounds of *GE* have been identified, and these are primarily phenolics (Ojemann et al., 2006; Li et al., 2016a; Zhan et al., 2016; Liu and Huang, 2017; Xu et al., 2019).

The molecular basis of the biosynthesis of bioactive ingredients in medicinal plants has attracted broad interest in recent years. Transcriptome sequencing and genome sequencing are common techniques used for this purpose (Han et al., 2016; Yang et al., 2016a; Saito, 2018; Trócsányi et al., 2020). Phenol-based compounds, especially gastrodin, are the dominant active components of *GE.* Pharmacokinetic studies show that these compounds play a vital role in the curative effect of *GE* (Ha et al., 2000; Hsieh et al., 2000; An et al., 2003; Yu et al., 2005; Kim et al., 2007; Makni et al., 2011; Tang et al., 2015; Tsai et al., 2016). By sequencing and analyzing the transcriptome of *GE*, the genes coding for enzymes that play a crucial role in the biosynthesis of gastrodin have been identified (Tsai et al., 2016; Yang et al., 2020; Chen and Mi, 2021). In addition, the genes associated with mannose-binding lectin antifungal proteins and the genes tightly related to the biosynthesis of the phenolics of *GE* have also been identified by transcriptome sequencing (Yang et al., 2020; Shan et al., 2021).

As mentioned above, the bioactive compounds related to healthful properties and their molecular basis of *GE* have been studied widely and understood deeply. However, which related to the nutritional properties of *GE* are still limited. As a result, our understanding of the molecular nutriology of *GE* is still far from comprehensive, which hinders the further utilization of this important functional food. So exploiting the abovementioned concerns is meaningful for developing the *GE* industry and a more comprehensive understanding of *GE* biology. For a complementary food, the type and content of amino acids and vitamins are undoubtedly important indicators of nutritional value. *GE* is a “polytypic species” composed of five forms according to morphological traits (Zhou et al., 1987). *Gastrodia. elata.f. elata* (*GEE*) and *Gastrodia. elata.f. glauca* (*GEG*) are two forms that were widely cultivated and commercially produced as medicinal materials or food ingredients. In this study, a UPLC-MS/MS platform was used to determine metabolome from four different tuber tissues of *GE*, each with three biological repeats. These consist of young and mature tubers of *G. elata f. elata* (*GEEy* and *GEEm*) and *G. elata f. glauca* (*GEGy* and *GEGm*), representing two common *GE* types and their different development stages. Meanwhile, the transcriptomes of the above samples were generated using a Hiseq 4000 sequencer. The metabolome and transcriptome of the above samples were performed as four pairwise (*GEEy* and *GEEm*, *GEGy* and *GEGm*, *GEEy* and *GEGy*, and *GEEm* and *GEGm*) analyses combined and separately. The specific aims of this study were to (1) reveal the similarities and differences in the types and contents of the metabolic components, especially the amino acid and vitamins between *GEE* and *GEG, and* their different development stages; (2) identify the functional genes involved in the biosynthesis and regulation of nutrient compounds, mainly amino acids, and vitamins, of *GE*. Overall, we want to provide insight into the nutritional properties of *GE and* its corresponding molecular basis, which will help deepen the biological understanding of *GE*.

## 2 Results

### 2.1 Pearson correlation assessment and principal component analysis of metabolomes and transcriptomes among samples

**Figure 1:**
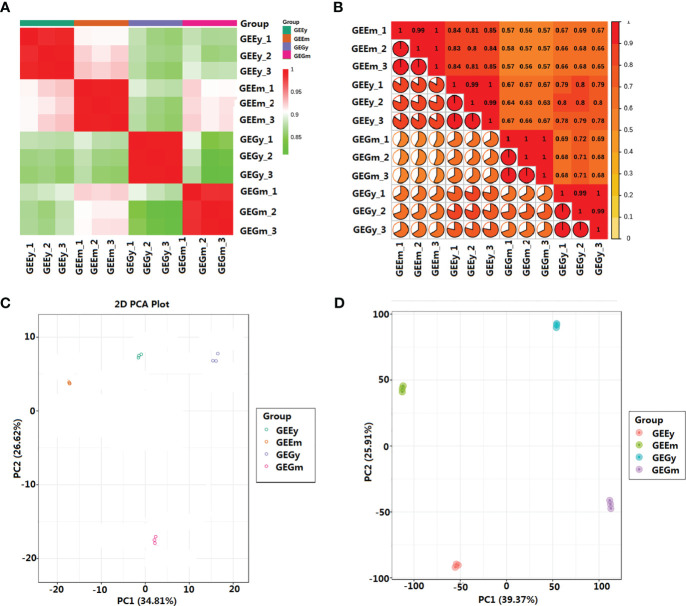
Relationships between the metabolites and transcripts of the samples. **(A)** Pearson’s correlation analysis of metabolites; **(B)** Pearson’s correlation analysis of transcripts; **(C)** Principal component analysis of metabolites; **(D)** Principal component analysis of transcripts. *GEEy* and *GEEm* indicate young and mature tubers of *G.elata.f.elata*, while *GEGy* and *GEGm* represent young and mature tubers *G.elata.f.glauca*, respectively.

Pearson’s correlation analysis showed that the metabolites of the four tuber tissues of *GE* were highly similar, with the lowest correlation coefficient (*r* ^2^) observed between samples being 0.81 (**Dataset S1**). The metabolite and transcript profiles in the sample repeats showed the highest correlation, with *r^2^* values reaching a minimum of 0.98 and 0.99, respectively, which is higher than those between non-biological repeat samples (**Figures 1A, B**; **Dataset S1**). The first and second principal components of metabolite and transcript profiles accounted for 34.81% and 39.73%, and 26.62% and 25.91% of the total variance, respectively. Meanwhile, the metabolome and transcriptome of biological repeats were distinctly clustered together compared to samples from different tissues (**Figures 1C, D**). The results of Pearson’s correlation analysis and principal component analysis (PCA) suggest that this study’s metabolite and transcript profile data are highly reliable.

### 2.2 Metabolites and transcripts profiles in four tuber tissues of GE

Three hundred forty-five unique metabolites were detected from twelve samples, including 75 types of amino acids and their derivatives (e.g., l-(+)-lysine, l-(+)-arginine, l-leucine, l-isoleucine, tryptophan, l-valine, l-(-)-threonine, l-phenylalanine, and l-methionine), and 13 kinds of vitamins, (e.g., nicotinamide, nicotinic acid, biotin, and riboflavin) (**Table 1**; **Dataset S2**). In addition, there are 45 Nucleotides and derivatives, 40 lipids, 18 organic acids, 39 types of phenolic acids, 34 types of alkaloids, 15 types of flavonoids, one tannin, five lignans and coumarin, three kinds of terpenoids also detected in young and mature tuber tissues of *GE* (**Table 1**; **Dataset S2**). Between 43,211,372 and 58,292,014 short raw reads were generated from the 12 samples using the Illumina Hiseq 4000 sequencer. After filtering out contamination, 42,333,902 to 57,576,240 clean reads, composed of 6.35 to 8.64 Gb, were obtained. More than 98% of the reads obtained for the samples were high-quality (> Q20) (**Table S1**). More than 90% of high-quality reads for each sample could be mapped on the released reference genome (**Table S2**). The mapping results identified 21,513 transcripts (genes), including 16,541 genes consistent with the sequences of the predicted protein-coding genes in the reference genome. In addition, 4,972 novel genes were also identified (**Table S3**).

**Table 1:** Counts of the metabolites in four tuber tissues of *G. elata* (*GE*).

Class	*GE*	*GEEy*	*GEEm*	*GEGy*	*GEGm*
Amino acids and derivatives	76	76	76	76	75
Nucleotides and derivatives	45	43	44	45	45
Lipids	40	40	40	40	40
Organic acids	18	18	18	18	18
Others	69	69	67	69	66
Phenolic acids	39	38	37	38	37
Flavonoids	15	15	15	13	14
Alkaloids	34	33	33	34	34
Terpenoids	3	3	3	3	2
Tannins	1	1	1	1	1
Lignans and Coumarins	5	3	5	3	3
Total	345	339	339	340	335

GE indicates Gastrodia elata; GEEy and GEEm indicate young and mature tubers of G. elata.f.elata (GEE), while GEGy and GEGm represent young and mature tubers of G. elata.f.glauca (GEG), respectively.

### 2.3 Functional annotation of metabolome and transcriptome and identification of genes related to amino acid and vitamin metabolism

**Figure 2:**
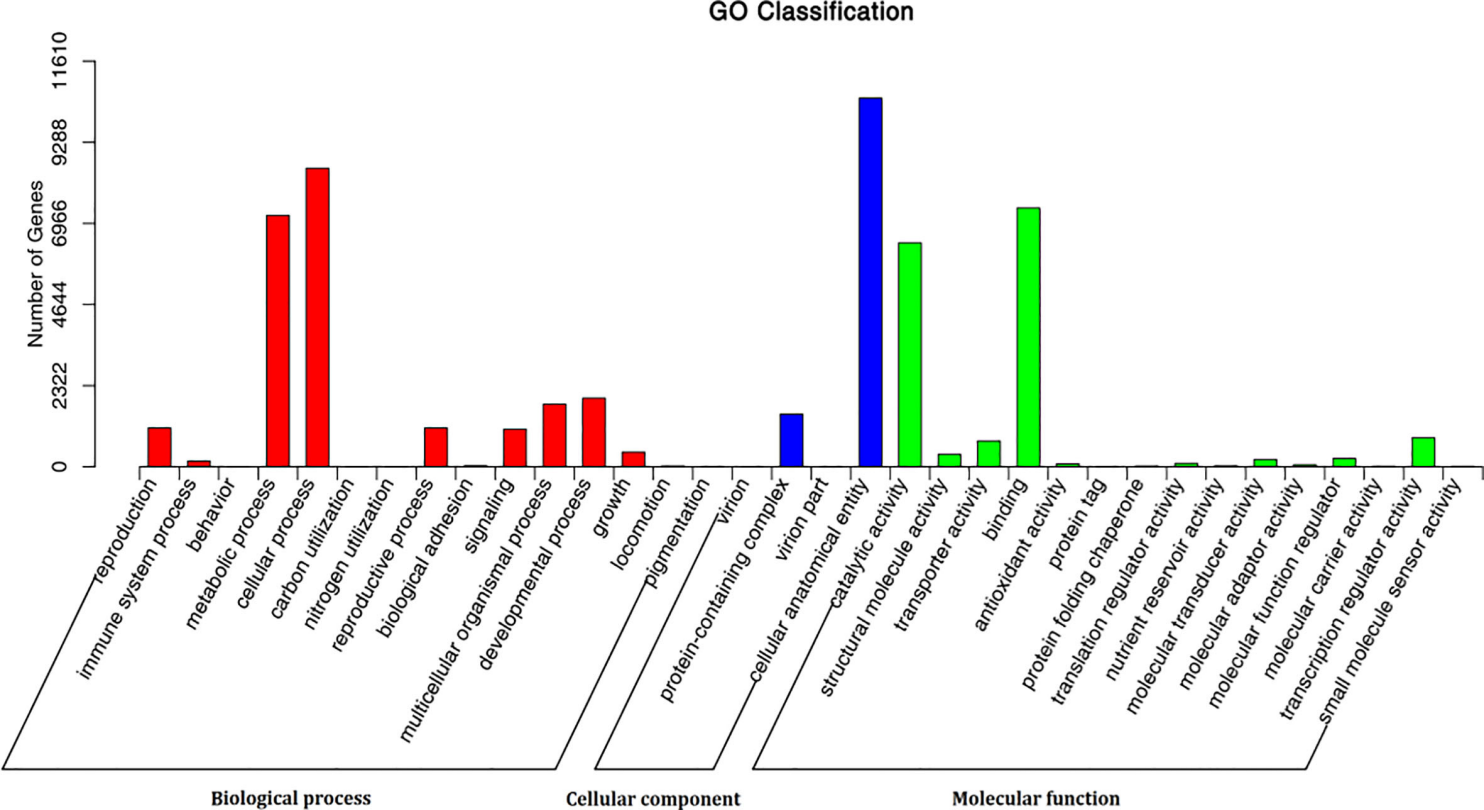
The classification statistics of GO annotation of genes of *Gastrodia elata*.

Of the 345 metabolites, 175 could be annotated by the Kyoto Encyclopedia of Genes and Genomes Database (KEGG) (https://www.kegg.jp/), but only 140 could be assigned to one or more of 82 specific metabolism pathways (**Figure S1**; **Dataset S3**). Of these, 126 (90%) were involved in various metabolic pathways, and 55 (39.29%) were involved in pathways related to the biosynthesis of secondary metabolites, including 25 metabolites were involved in pathways related to the biosynthesis of amino acids, four, three, five, seven, seven and two were related to the pathways “thiamine metabolism”, “riboflavin metabolism”, “vitamin B6 metabolism”, “nicotinate and nicotinamide metabolism”, “pantothenate and CoA biosynthesis” and “biotin metabolism” respectively (**Figure S1**; **Dataset S3**). Of the 21,513 identified genes, 16,453, (76.48%) could be annotated using at least one of the Nr, SwissPort, KOG, GO and KEGG databases (**Table S3**; **Dataset S4**). Of the 16,426 genes annotated using the Nr database, 13,585 (82.68%) were matched using the homologous sequences of *Dendrobium catenatum*, *Phalaenopsis* equestris, and *Apostasia shenzhenica*, which are also types of orchids (**Table S3**; **Figure S2**; **Dataset S4**). Of 12,223 GO annotated genes, the GO terms mainly fall into “cellular process” and “metabolic process” of biological process, and “cellular anatomical entity” of cell component, and “binding”, “catalytic activity” of molecular function category (**Figure 2**; **Dataset S4**). A total of 5,720 genes were assigned to 138 pathways using KEGG annotation (**Figure S3**; **Dataset S4**). Of these, 17 genes were identified to code for eight enzymes (lysC, asd, hom, dapA, dapB, dapL, dapF, and lysA) involved in the lysine biosynthesis, which uses l-aspartate as the initial substrate. LysA, coded by the gene Gene-tia007535, played a direct role in the biosynthesis of lysine and was identified in this analysis (**Figure S4**; **Dataset S4**). 24 genes were found to involve in the pathway of histidine metabolism, including enzyme his G coding gene (gene-tia009269), his E coding genes (e.g., gene-tia012550, his I coding genes (e.g., gene-tia009135), his F coding genes (e.g., gene-tia011633), his B coding gene (gene-tia009637), his C coding genes (gene-tia009637), and his D coding genes (e.g., gene-tia00517), which constitutes a complete metabolic pathway and catalyzes the substrate PRPP into l- histidine (**Figure S5**; **Dataset S4**). Forty-five genes (e.g., gene-tia006823, gene-tia009761, gene-tia011646) were identified to be related to phenylalanine, tyrosine, and tryptophan biosynthesis. These separately coded whole set enzymes (aroG, aroB, aroD, aroE, aroK, aroA, aroC, TRP3, trpD, TRP1, trpA, chorismate mutase, PAT, AAT, tyrAa, ADT, PDT, eta) that catalyze the substrate d-erythrose 4-phosphate into above three amino acids (**Figure S6**; **Dataset S4**). Among the 23 genes encoding 11 enzymes (SIR2, NMNAT, URH1, NAPRT1, 5`nucleotidase, nadD, CD203, nudC, nadE, nadD, nadK, nadB, and NADK) that are involved in the nicotinate and nicotinamide metabolism pathway, URH1 (which is coded for by gene-tia004446 and gene-tia004985) and NAPRT1 (which is coded for by gene-tia008609) are key enzymes for the mutual transformation of nicotinate and nicotinate D ribonucleotide. The enzyme punA also catalyzes this mutual transformation, but no gene was found that codes for this enzyme (**Figure S7**; **Dataset S4**). Among 14 genes involved in the pathway of thiamine metabolism, the gene-tia010644 and gene-tia016175 codeTHI20, gene-tia010644 and gene-tia016175 code thiE, gene-tia002595 and gene-tia003843 code rsgA, and these three enzymes can catalyze the 4-amino-5-hydroxymethyl-2-methylpyrimidine phosphate into thiamine) (**Figure S8**; **Dataset S4**).

### 2.4 Metabolite accumulation similarity and differences among samples

**Figure 3:**
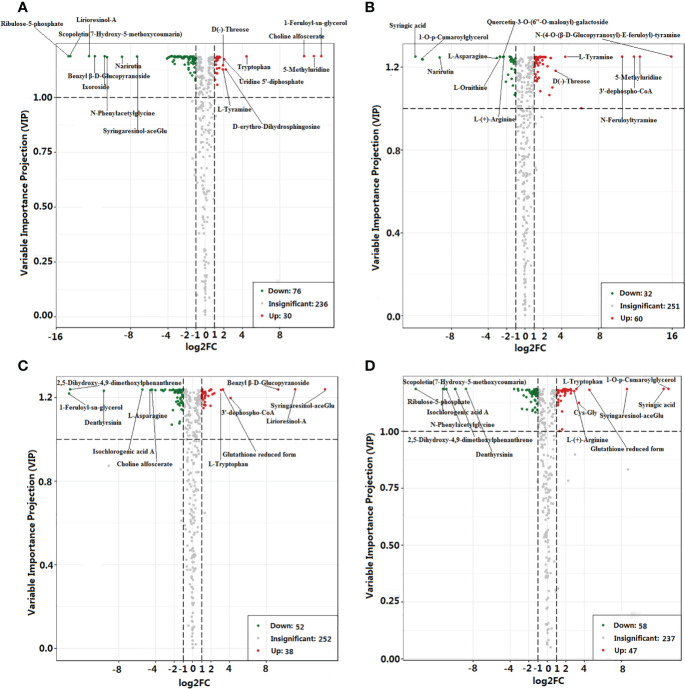
Statistics of different and stable accumulation of metabolites between comparisons. **(A)** *GEEm vs. GEGm*; **(B)** *GEEy vs. GEEm*; **(C)** *GEEy vs.* **(D)** *GEGy*; *GEGy vs. GEGm*. *GEEy* and *GEEm* indicate young and mature tubers of *G. elata.f.elata*, while *GEGy* and *GEGm* indicate young and mature tubers of *G. elata.f.elata*.

Significant differences in the accumulation of metabolites (DAMs) were observed between *GEEm* and *GEGm for* 106 metabolites, *GEEy* and *GEGy for* 90 metabolites, and 36 metabolites in the above two pairwise samples (**Figures 3A, B**; **Dataset S5**). Of these, nine were up-regulated, including one amino acid (l-tyramine) and twenty-seven metabolites, including five amino acids and their derivatives (l-asparagine, l-ornithine, l-(+)-arginine l-(-)-tyrosine and 2,6-diaminooimelic acid) were down-regulated based on pairwise analysis of *GEEm vs. GEGm*, and *GEEy vs. GEGy* (**Dataset S5**). 92, 105, and 33 DAMs were identified from pairwise studies of *GEEy vs. GEE*m, *GEGy vs. GEGm, and* both *GEEy vs. GEE*m and *GEGy vs. GEGm* (**Figures 3C, D**; **Dataset S5**). Nine were down-regulated, including one amino acid derivative (e.g., alanylleucine). Twenty-four metabolites, including nine amino acids and their derivatives (l-(-)-threonine, α-aminocaproic acid, l-isoleucine, l-allo-isoleucine, l-leucine, glutamic acid, Cys-Gly, l-tryptophan, glutathione reduced form) were up-regulated based on pairwise analysis of *GEEy vs. GEEm* and *GEGy vs. GEGm* (**Dataset S5**). According to four comparisons, most metabolites accumulate with non-significant differences between samples (*GEEy vs. GEEm*, *GEGy vs. GEGm*, *GEEy vs. GEGy* and *GEEm vs. GEGm*). 139 metabolites, including 22 amino acids and derivatives (e.g., l-phenylalanine, l-methionine, histamine, l-2-chlorophenylalanine, d-serine, n-α-acetyl-l-glutamine), 10 vitamins (e.g., nicotinamide, thiamine, and biotin) and 15 alkaloids (e.g., agmatine, spermidine, and spermine) contents were found to be similar in all four samples (**Dataset S5**). Ten metabolites, including l-asparagine and gastrodin, are DAMs based on all the above four pairwise analyses (**Dataset S5**).

### 2.5 Differentially expressed genes among samples and these related to amino acids and vitamin metabolism

**Figure 4:**
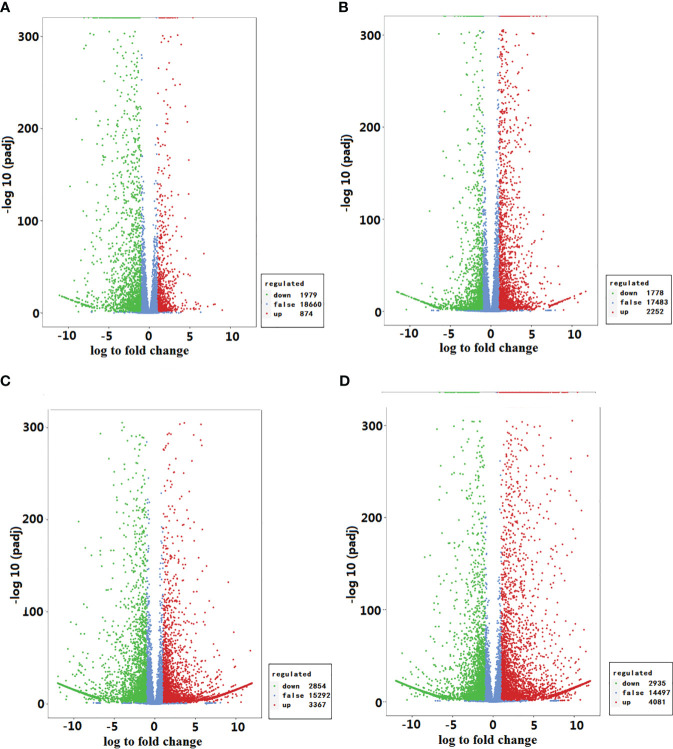
Statistics of different and stable expressions of genes between different comparisons. **(A)** *GEEy vs. GEEm*; **(B)** *GEEy vs. GEGy*; **(C)** *GEEm vs. GEGm*; **(D)** *GEGy vs. GEGm GEEy* and *GEEm* indicate young and mature tubers of *G. elata.f.elata*, while *GEGy* and *GEGm* represent young and mature tubers *G. elata.f.glauca*, respectively.

There were 2853 differentially expressed genes (DEGs), 1979 down-regulated and 874 up-regulated, in *GEEy* compared with *GEEm*, and 4030 DEGs, 1778 down-regulated and 2252 up-regulated, in *GEGy* when compared with *GEGm*. Based on pairwise analyses, 153 down-regulated and 76 up-regulated DEGs appeared between *GEEy vs. GEEm* and *GEGy vs. GEGm* (**Figures 4A, B**; **Dataset S6**). The DEGs from the two pairwise analyses are significantly enriched in several KEGG pathways, including starch and sucrose metabolism, flavonoid biosynthesis, and phenylpropanoid biosynthesis pathways (**Figure S9**; **Dataset S7**). Of these DEGs from the two pairwise analyses, 18 (e.g., gene-tia002357, gene-tia002852, gene-tia003785, gene-tia004085, gene-tia005120) were identified to be involved in pathways biosynthesis of amino acids, one (gene-tia010644), two (gene-tia001539, gene-tia003140), two (gene-tia014555, gene-tia000824), one (gene-tia004985), two (gene-tia009761, gene-tia002852) and one (gene-tia009822) were identified to be related to thiamine metabolism, riboflavin metabolism, vitamin B6 metabolism, nicotinate, and nicotinamide metabolism, pantothenate and CoA biosynthesis and biotin metabolism, respectively (**Dataset S7**). There were 6221 DEGs, 2854 down-regulated and 3367 up-regulated, found in *GEEy* compared with *GEGy*, and 7016 DEGs, 2935 down-regulated and 4081 up-regulated, found in *GEEm* when compared with *GEGm*. Among these DEGs, 3766 appeared in both pairwise analyses (**Figures 4C, D**; **Dataset S6**). The DEGs between *GEE*y and *GEE*m and between *GEG*y and *GEG*m are significantly enriched in several KEGG pathways, including galactose metabolism, flavonoid biosynthesis, and phenylpropanoid biosynthesis (**Figure S9**; **Dataset S7**). Among these DEGs from the two pairwise analyses, 28 (e.g., gene-tia001033, gene-tia001508, gene-tia001739, gene-tia002208, gene-tia004922) were related to the biosynthesis of amino acids, two (gene-tia003026, gene-tia014391), four (gene-tia003899, gene-tia001539, gene-tia001540, gene-tia003140), three (gene-tia015010, gene-tia000824, gene-tia000825), three (gene-tia009709, gene-tia011772, gene-tia01282), four (gene-tia009761, gene-tia001787, gene-tia012005, gene-tia005298) and five (gene-tia017675, gene-tia017698, novel.2534, novel.2739, gene-tia015278) were identified to be related to thiamine metabolism, riboflavin metabolism, vitamin B6 metabolism, nicotinate, and nicotinamide metabolism, pantothenate and CoA biosynthesis and biotin metabolism, respectively (**Dataset S6**).

### 2.6 Correlation and network expression of DAMs and DEGs

**Figure 5:**
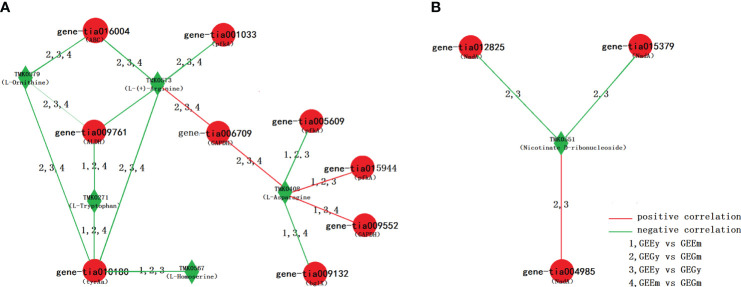
Network of DEG and DAM. **(A)** DEG and DAM pairs based on three comparisons, which is involved in the pathway of amino acid biosynthesis; **(B)** DEG and DAM pairs based on two comparisons, which is involved in the pathway of nicotinate and nicotinamide metabolism.

Many pairs of DEGs and DAMs, based on the comparisons of *GEEy* and *GEGy*, *GEEm* and *GEGm*, *GEEy* and *GEEm*, and *GEGy* and *GEGm*, were found to be involved in the same pathways and showed a significant relationship (**Dataset S8**). 732, 76, and one pair appeared in two, three, and four comparative pairwise analyses. It showed the same correlation trend, implying a stable one-way or two-way direction in the positive or negative expression regulation between them in the tubers of *GE* (**Dataset S9**). Of 76 pairs based on three comparisons, 16 DEG-DAM pairs consisted of five metabolites (TMK0579:l-ornithine, TMK0408:l-asparagine, TMK0573:l-(+)-arginine; TMK0271:l-tryptophan; TMK0567:l-homoserine) and nine genes (gene-tia001033, gene-tia005609 and gene-tia015944 coding pfkA, gene-tia009552 and gene-tia006709 coding GAPDH, gene-tia009132 coding bglX, gene-tia009761 coding ALDH, gene-tia010180 coding tyrAa, gene-tia016004 coding ABC) were identified to be related to the biosynthesis of amino acid, four pairs, including gene-tia006709-TMK0408, gene-tia006709-TMK0573, gene-tia009552-TMK0408 and gene-tia015944-TMK0408 show positive correlation and other 12 pairs (e.g., gene-tia001033-TMK0579, gene-tia005609-TMK0408, gene-tia009761-TMK0271, gene-tia010180-TMK0567, gene-tia016004-TMK0573) show a negative correlation (**Figure 5A**; **Dataset S9**). Of 732 pairs based on two comparisons, Three pairs of DEG-DAM consisted of one metabolite (TMK0551:nicotinate d-ribonucleoside) and three genes (gene-tia015379 and gene-tia012825 code NadA and gene-tia004985 coding uridine nucleosidase) based on two comparisons were found to involved in nicotinate and nicotinamide metabolism, pair of gene-tia004985 and TMK0551 shows a positive correlation, pairs of gene-tia015379 and TMK0551, and gene-tia012825 and TMK0551 show a negative correlation (**Figure 5B**; **Dataset S9**).

## 3 Discussion

### 3.1 GE is an excellent complementary food, but it should not be consumed excessively

A total of 345 metabolites were found in *GEEy, GEEm, GEGy*, and *GEGm*. Relative to other plant tissues, the number of metabolites found in the tuber tissue of *GE* is lower when determined using the same method. For example, there are approximately 600 metabolites found in the leaf, flower, root, and fruit of *Eriobotrya japonica* (Wang, 2021), and 661 are found in the different development stages of *Chrysanthemum morifolium* (Wang et al., 2019). In chestnuts (*Castanea mollissima* Bl.), 611 metabolites were found to have varying degrees of calcification (Xiao et al., 2021). In the young shoots of Albino Tea cultivars, 839 metabolites have been found (Wang et al., 2020), with 499 metabolites found in *Allium fistulosum*–*A. cepa* (Abdelrahman et al., 2019), and 637 were found in different tissues of *Salsola collina* Pall (Li et al., 2021). We speculate that this is because *GE* adopts a heterotrophic lifestyle (Park and Lee, 2013), and some metabolic pathways in autotrophic plants fail to function in *Gastrodia elata*, so the corresponding metabolites are also reduced. Seventy-six types of amino acids, including eight kinds of amino acids (l-valine, l-(+)-lysine, l-leucine, l-isoleucine, tryptophan, l-(-)-threonine, l-phenylalanine, and l-methionine) that the human body cannot synthesize and that must be supplemented from the diet, and 13 type vitamins were found in this study, which revealed that *GE* contained excellent auxiliary ingredients. Besides 39 phenolic acids and 15 flavonoids containing potential healthful ingredients (e.g., gastrodin, cinnamic acid, narirutin, tangerine), 34 alkaloids were also identified in the tuber of *GE*, which contain some higher accumulated metabolites, such as choline and spermine. Choline is a very effective healthful-promoting compound (Wecker et al., 1982; Leermakers et al., 2015), and spermine is a necessary component of all vertebrate cells, but toxic effects will be produced if it accumulates in cells (Seiler et al., 2000). This investigation demonstrates that the spermine contents in all four samples were relatively higher. As a result, the *GE* might not be suitable for excessive food consumption.

### 3.2 *GEGm* excels over *GEEy*, *GEEm*, and *GEGy* for supplementing amino acids

In total, 335 to 339 metabolites were identified from young and mature tubers of *GEE* and *GEG*, respectively, suggesting slight variations in the metabolites among different *GE* varieties and the various development stages of *GE.* This is similar to the results of Xiong et al. (2014) and Yang et al. (2016b). The quality of *GE* is mainly reflected in the content of specific nutritional and medicinal components. The types and abundance of amino acids are important indicators of the nutritional quality of *GE* (Lei et al., 2015). There are conflicting views on whether *GE*, *GEG* or *GEE* present better medicinal and nutritional qualities (Wu et al., 2011; Tan et al., 2012; Li et al., 2016a; Yang et al., 2016b; Wang et al., 2018). In this study, nine different amino acids, including glutamic acid, Cys–Gly, l-tryptophan, and glutathione (reduced form), showed higher content in mature tubers than in young tubers. Only one amino acid, alanyl-leucine, showed lower abundance in mature tubers than in young tubers. Therefore, if the nutrient quality of *GE* is judged based on the content of amino acids, the mature tuber of *GEGm* excels over that of the other tuber samples. Another key nutrient element in supplementary food is vitamins. Of identified 13 type vitamins in four samples of *GE*, 10 are no-DAM metabolites based on four comparisons, only nicotinic acid in *GEGm* is higher than that of *GEEm*, and nicotinate d-ribonucleoside in *GEGy* is higher than that of *GEEy*, nicotinate d-ribonucleoside and riboflavin in *GEGy* are higher than that in *GEGm*. These results show that *GEG*, especially *GEGy*, has a slight advantage over *GEE*.

### 3.3 First insights into the molecular basis of amino acids and vitamins that related to the nutritional properties

The main objective of pharmacognosy is to uncover the molecular basis and genetic mechanism of bioactive ingredients with potential health values in medicinal plants. In recent years, attempts have been made to uncover the genetic mechanism of phenols, including gastrodin found in *GE*, by comparing the transcriptomes from different developmental stages of *GE* tubers. For example, Tsai et al. (2016) identified two unigenes encoding glycosyltransferase and monooxygenase as the molecular basis of gastrodin biosynthesis in the tubers of *GE*. Genes code eleven key enzymes, including CM, PDT, PAL, 4CL, CHS, F5H, F3’M, C4H, CCR, and CAD, are involved in the phenylpropanoid metabolic pathway and are most likely responsible for the molecular mechanism of the medicinal quality formation of *GE*, were detected (Wen et al., 2017). Seventy-six candidate genes encoding eight key enzymes involved in the *GE* phenolics biosynthesis pathway were identified (Shan et al., 2021). Using co-expression analysis, Chen and Mi (2021) showed that 4-hydroxybenzyl alcohol mainly originates from phenylalanine metabolism. Polysaccharides are another group of critical active ingredients found in *GE* (Zhu et al., 2019) and have been found to exhibit various activities, including anti-cancer (Zhao et al., 2005), antioxidation, and anti-aging (Liu and Mori, 1992; Xie et al., 2010) activities; immunomodulatory effects (Li et al., 2016b); neuroprotection (Zhou et al., 2018); and cardiovascular system activities (Kim et al., 2012). Previous studies show that the polysaccharide composition of *GE* included xylose, glucose, galactose, rhamnose, and mannose, with glucose accounting for the vast majority (Zhao et al., 2005; Qiu et al., 2007; Li et al., 2008; Chen et al., 2011; Lee et al., 2012; Ming et al., 2012; Bao et al., 2017). These monosaccharides are linked by 1→6 and 1→4 glycosidic bonds, or 1→2 glycosidic bonds (Zhou et al., 2009), with 1→4 glycosidic bonds being the most common (Zhao et al., 2005; Qiu et al., 2007; Ming et al., 2012). Previous studies on *GE*’s bioactive substances and corresponding molecular basis mainly focused on the health-promoting components.

Until now, investigations on nutrient components and molecular mechanisms underlying *GE* have been limited. Under the new background that *GE* was officially listed drug and food source by the Chinese government, it is particularly urgent to reveal the molecular basis related to the nutritional characteristics of *GE*. Here, we detected 76 different amino acids and their derivatives, 13 vitamins, and some other metabolism endow the nutritional properties. We also identified many genes that code enzymes responsible for the biosynthesis or metabolism of some amino acids (lysine, histidine, phenylalanine, tyrosine, tryptophan) and vitamins (nicotinate, nicotinamide, thiamine). Meanwhile, comparing the integrative analyses of the metabolomes and transcriptomes of different tuber samples pairwise, many DEG and DAM pairs were annotated into the same metabolism pathways. Some of these pairs exhibit a strong positive or negative correlation with a similar trend, indicating that there is a consistent positive or negative regulation between them. Additionally, it was found that certain vitamins and amino acids directly promote or inhibit the expression of the genes involved in the biosynthesis of these metabolites.

## 4 Conclusions

A total of 345 metabolites and 21,513 transcripts were detected in *GEEy*, *GEEm*, *GEGy* and *GEGm.* These metabolites show that fresh *GE* tissues make a great supplemental diet since they contain 76 amino acids and their derivatives, which contain all of the human necessary amino acids (such as l-(+)-lysine and l-leucine) and 13 vitamins. However, consuming too much *GE* could be dangerous because of its high spermine content. Of *GEEy*, *GEEm*, *GEGy*, and *GEGm*, *GEGm* has higher amino acid ingredients. Meanwhile, some genes metabolizing some amino acids and vitamins were identified. There were also a few whose up- or down-regulated expression would considerably encourage or prevent the accumulation of various vitamins and amino acids. In general, the data and analysis in this work represent a significant step forward in understanding the molecular basis, genetic basis, and regulatory mechanism of GE nutritional characteristics, which will aid in the rational consumption of *GE* industry and deepen our understanding of *GE* biology.

## 5 Materials and methods

### 5.1 Plant material sampling and tissue pretreatment

Young (length=~3cm, diameter=~2cm, the terminal bud is not apparent) and mature (length=~10cm, diameter=~25cm, the terminal bud is obvious) tubers of a GEG strain, grown at an altitude of around 2300 meters, were taken from Xiaocaoba town, Yiliang county, Yunnan province. The young (length=~3cm, diameter=~2cm, the terminal bud is not obvious) and mature (length=~12cm, diameter=~20cm, the terminal bud is obvious) tuber of a GEE strain, grown at an altitude of around 1500 meters, were taken from Niuzhuang town, Yiling county, Hubei Province. Both strains were cultivated in sandy soil rich in humus under broad-leaved forests before sampling. Both sample collection sites belong to the humid climate zone of the central subtropical zone, with mountainous terrain. The average annual temperature and precipitation in Xiaocaoba are 15°C and 1100 mm, while in Niuzhuang they are 10°C and 1600 mm, respectively. In order to avoid contamination from the tissues of symbiotic Armillaria mellea, the tubers were stripped of the surface cortex before the tissue within the epidermal layer was freeze-dried and ground into powder. One hundred milligrams of powder from each sample was weighed and used for total RNA extraction. Then, another 100 mg of powder from each sample was dissolved in 0.6 ml of 70% methanol extract. The dissolved samples were refrigerated overnight at 4°C and centrifuged at 10,000 rpm. The supernatant was filtered with a microporous membrane (0.22 μm pore size) and stored in an injection bottle, ready for UPLC-MS/MS analysis. Three biological repeats were carried out for each tissue sample. The remaining sample tissues not utilized in this experiment were kept at Kaili University’s -80°C refrigerator.

### 5.2 Metabolite data acquisition using UPLC-MS

Ultra-high Performance Liquid Chromatography (UHPLC) (Shim-pack UFLC SHIMADZU CBM30A; https://www.shimadzu.com.cn/) and tandem mass spectrometry (Applied Biosystems 4500 qtrap; http://www.appliedbiosystems.com.cn/) were used to collect metabolite data. The UHPLC conditions were as follows: chromatographic column (Waters ACQUITY UPLC HSS T3 C18 1.8 μm, 2.1 mm * 100 mm); mobile phase A (ultra-pure water with 0.04% acetic acid); mobile phase B (acetonitrile with 0.04% acetic acid); elution gradient (the starting concentration of phase B was 5%; phase B was increased linearly to 95% within 10.00 min and maintained at 95% for 1.0 min; phase B decreased to 5% at 11.00–11.10 min and maintained at 5% for 14 min. Flow rate = 0.35 ml/min; column temperature = 40°C; and injection volume = 4 μL). The mass spectrum conditions were set as follows: the temperature of electrospray ionization was set to 550°C, the voltage was set to 5500 V, the curtain gas pressure was fixed at 30 psi, and the parameter of collision-activated dissociation was set to high. In the triple quadrupole (QQQ), each ion pair was scanned and detected according to the optimized declustering potential and collision energy (Chen et al., 2013).

### 5.3 Qualitative and quantitative analysis of metabolites

The self-built metware database (MWDB) by Metware Biotechnology Co., Ltd. (Wuhan, China) was used to qualitatively analyze the metabolites based on secondary spectrum information. Repeated signals, including NH4^+^ ion, Na^+^ ion and K^+^ ion, and the repeated signal of fragment ions for larger molecular weight substances were removed. Metabolite quantification was performed via multiple reaction monitoring mode analysis using triple quadrupole mass spectrometry (Fraga et al., 2010).

### 5.4 Metabolite data processing and analysis

The mass spectrum data were handled using Software Analyst 1.6.3. PCA, clustering, repetitive correlation, and differential expression analysis were performed using the MetaboAnalystR software package (Chong and Xia, 2018). The KEGG database was used to annotate and display the biosynthetic pathways of different metabolites.

### 5.5 RNA extraction, library construction, and sequencing

The total RNA from tuber tissues was extracted using an RNA kit (Thermo Fisher Scientific Inc, Waltham, MA, USA) following the guidelines provided by the manufacturer. The concentration and quality of the extracted total RNA were determined using Nanodrop 2000 (Thermo Fisher Scientific, USA). The integrity of the RNA samples was evaluated by agarose gel electrophoresis. The total RNAs with concentrations ≥ 300ng/μL, OD260/280 = 1.8–2.0 were used to construct the sequencing library as follows: (1) mRNA was extracted from the total RNA using Oligo (dT) magnetic beads; (2) approximately 300 bp length mRNA fragment sequences were obtained, and first strand of cDNA was synthesized; (3) the RNA strand was degraded by RNaseH before the second strand of cDNA was synthesized with dNTPs with the help of a DNA polymerase I system; (4) purified double-stranded cDNA gel electrophoresis was performed, and the end of the cDNA was repaired; (5) cDNA was screened for 250–300 bp lengths, and polymerase chain reaction (PCR) products were refined using Ampure XP beads; (6) and finally, the library was obtained. More than 6G of raw data per library were generated using a Hiseq 4000 sequencer (Illumina, San Diego, CA, USA).

### 5.6 RNA sequencing data filtering and statistics

The raw sequencing data were filtered by removing the reads containing an adaptor sequence, paired-end reads with an N content exceeding 10% of the length proportion in either single read and low-quality (≤ 5) base numbers exceeding 50% of the length proportion in either single read. The remaining high-quality clean data were used for analyses. Sequencing reads the sequencing error rate, the Q20 ratio, Q30 ratio, and GC content of the raw and clean data outputs were counted using a custom Pearl script developed by Maiwei Co., Ltd (https://www.metware.cn/). The raw transcriptome data of twelve samples have been deposited in GenBank (https://www.ncbi.nlm.nih.gov/) (Accessible ID: PRJNA825667).

### 5.7 Clean RNA sequencing data mapping and gene expression statistics

Clean RNA reads with > Q20 for *GEEm*, *GEEy*, *GEGm*, and *GEGy* were mapped onto the assembled reference genome of *G. elata Bl.f.elata* assembled (Wang and Shahid, 2023) using HISAT v2.0.5 (https://arc-ts.umich.edu/software-item/hisat2/) to generate reference transcripts. Subsequently, the clean reads were mapped onto the transcripts using Bowtie2 v2.3.4.1 (https://wiki.rc.usf.edu/index.php/Bowtie2#Version). If a transcript sequence mapped onto the predicted protein-coding region of the reference genome, this was regarded as an existing gene; otherwise, the sequence was regarded as a new gene. Based on the mapping results produced by Bowtie2, the number of reads mapped on each transcript in each sample was counted. Then, the FPKM (fragments per kilobase per million bases)-transformed results were generated using RSEM software (Li and Dewey, 2011). The expression abundance of protein-coding genes or their transcripts was obtained. The PCA and Pearson’s correlation analysis of the biological repeats based on total gene expression was performed using the R software package (https://www.r-project.org/). DEGs between sample groups were analyzed using the software DESeq2 (Love et al., 2014).

### 5.8 Functional annotation of transcriptome and DEGs by KEGG and GO enrichment analysis

The transcriptome, including DEGs, were annotated for molecular function using various databases, including Nr (https://www.ncbi.nlm.nih.gov/refseq/), SwissPort (http://www.ebi.ac.uk/swissprot/), KOG (https://www.ncbi.nlm.nih.gov/COG/), GO (http://geneontology.org/), and KEGG using BLAST v2.10.0 software (https://www.ncbi.nlm.nih.gov/books/NBK131777/) with 1e^-5^ as the threshold of the e-value. Based on the GO and KEGG annotation, GO enrichment and KEGG enrichment analyses of DEGs were further carried out using GOseq (Young et al., 2010) and KOBAS (Mao et al., 2005) software, respectively.

### 5.9 Combined analysis of transcriptomes and metabolomes

Firstly, a combined KEGG annotated pathway was produced according to this experiment’s differential metabolite analysis results and the transcriptome differential gene analysis results. In other words, the differential genes and metabolites of the same paired sample groups for comparison were simultaneously mapped to the KEGG pathway map to understand the expression relationship between metabolites and genes. Secondly, the cor program in R software was used to estimate Pearson’s correlation coefficient of differential genes and differential metabolites. Finally, the metabolites and genes with Pearson correlation coefficients greater than 0.8 in each different group in each pathway were analyzed by canonical correlation analysis using R software (https://www.r-project.org/).

## Data availability statement

The datasets presented in this study can be found in online repositories. The names of the repository/repositories and accession number(s) can be found at the NCBI, accession number: PRJNA825667.

## Author contributions

YW designed the experiments and collected samples. YW and MS analyzed data and wrote the paper. All authors contributed to the article and approved the submitted version.

## Supplementary material

Supplementary Data Sheet 7 List of differentially expressed genes (DEGs).

Supplementary Data Sheet 9 Network correlation of DEGs and DAMs involved in same KEGG pathways.
