# Supplementary material for: Insights into the nutritional properties and molecular basis of biosynthesis of amino acids and vitamins of Gastrodia elata offered by metabolomic and transcriptomic analysis
Source: Front Plant Sci. 2023 Jun 26;14:1183139. doi: 10.3389/fpls.2023.1183139 (PMC10331839; doi:10.3389/fpls.2023.1183139)
Supplement: Supplementary Data Sheet 7 — List of differentially expressed genes (DEGs). [file DataSheet_1.zip › Table 1 - 2023-06-07T162207.154.DOCX]

**Insights into the nutritional properties and molecular basis of biosynthesis of amino acids and vitamins of *Gastrodia elata* offered by metabolomic and transcriptomic analysis**

Yunsheng Wang, Muhammad Qasim Shahid

**Supplementary Figures and Tables**

Table S1 Summary of transcriptome data and mapped clean reads

| Sample | Raw Reads | Clean Reads | Clean Base (Gb) | Q20(%) | Q30(%) | GC (%) |
| --- | --- | --- | --- | --- | --- | --- |
| *GEEm1* | 47983570 | 47241352 | 7.09 | 98.15 | 94.46 | 47.3 |
| *GEEm2* | 58292014 | 57576240 | 8.64 | 98.18 | 94.48 | 47.23 |
| *GEEm*3 | 45079952 | 44455630 | 6.67 | 98.22 | 94.44 | 47.12 |
| *GEEy*1 | 43211372 | 42333902 | 6.35 | 98.22 | 94.71 | 48.36 |
| *GEEy*2 | 49155544 | 48141374 | 7.22 | 98.22 | 94.68 | 48.82 |
| *GEEy*3 | 50492760 | 49087062 | 7.36 | 98.23 | 94.68 | 48.8 |
| *GEGm*1 | 49040256 | 48264950 | 7.24 | 98.26 | 94.8 | 49.11 |
| *GEGm*2 | 57221874 | 56280430 | 8.44 | 98.27 | 94.75 | 47.27 |
| *GEGm*3 | 51476390 | 50754280 | 7.61 | 98.29 | 94.83 | 48.29 |
| *GEGy*1 | 46771422 | 46054372 | 6.91 | 98.31 | 94.81 | 47.41 |
| *GEGy*2 | 43905288 | 43445026 | 6.52 | 98.34 | 94.9 | 47.16 |
| *GEGy*3 | 49568912 | 48958566 | 7.34 | 98.41 | 95.14 | 48.23 |

Note: *GEEy* and *GEEm* indicate young and mature tubers of *G. elata.f.elata*, while *GEGy* and *GEGm* represent young and mature tubers of *G. elata.f.glauca*, respectively.

Table S2 Mapping statistics of transcriptome sequencing reads against the reference genome

| Sample | Number of mapped reads (%) | Unique mapped reads (%) | Multi-mapped reads (%) |
| --- | --- | --- | --- |
| *GEEm1* | 45398285 (96.10%) | 43478085 (92.03%) | 3939276 (4.06%) |
| *GEEm2* | 55389587 (96.20%) | 52962189 (91.99%) | 5001381(4.22%) |
| *GEEm*3 | 42796329 (96.27%) | 40978228 (92.18%) | 3725403 (4.09%) |
| *GEEy*1 | 40946967 (96.72%) | 38264449 (90.39%) | 4699084 (6.34%) |
| *GEEy*2 | 46534443 (96.66%) | 43523339 (90.41%) | 5219681 (6.25%) |
| *GEEy*3 | 47523920 (96.82%) | 44388768 (90.43%) | 5522337 (6.39%) |
| *GEGm*1 | 43928545 (91.02%) | 40250942 (83.40%) | 8761656 (7.62%) |
| *GEGm*2 | 51227824 (91.02%) | 46802652 (83.16%) | 10688988 (7.86%) |
| *GEGm*3 | 46301445 (91.23%) | 42413022 (83.57%) | 9183200 (7.66%) |
| *GEGy*1 | 41765052 (90.69%) | 39133768 (84.97%) | 5203022 (5.71%) |
| *GEGy*2 | 39413160 (90.72%) | 37047406 (85.27%) | 4598475 (5.45%) |
| *GEGy*3 | 44368288 (90.62%) | 41585180 (84.94%) | 5476791 (5.68%) |

Note: *GEEy* and *GEEm* indicate young and mature tubers of *G. elata.f.elata*, while *GEGy* and *GEGm* represent young and mature tubers of *G. elata.f.glauca*, respectively.

Table S3. Transcripts count and their functional annotation

| Items | Counts |
| --- | --- |
| Total genes | 21,513 |
| Novel genes | 4,972 |
| Total annotated gene | 16,453 |
| Nr annotation genes | 16,431 |
| SwissPort annotation genes | 6,938 |
| KOG annotation genes | 9,956 |
| GO annotation genes | 12,223 |
| KEGG annotation genes | 5,953 |
| KEGG Pathway annotation genes | 5,959 |
| Pfam | 5,431 |
| Tremble | 16,373 |
| Transcription factor | 1,268 |
| Un-annotated genes | 5,060 |


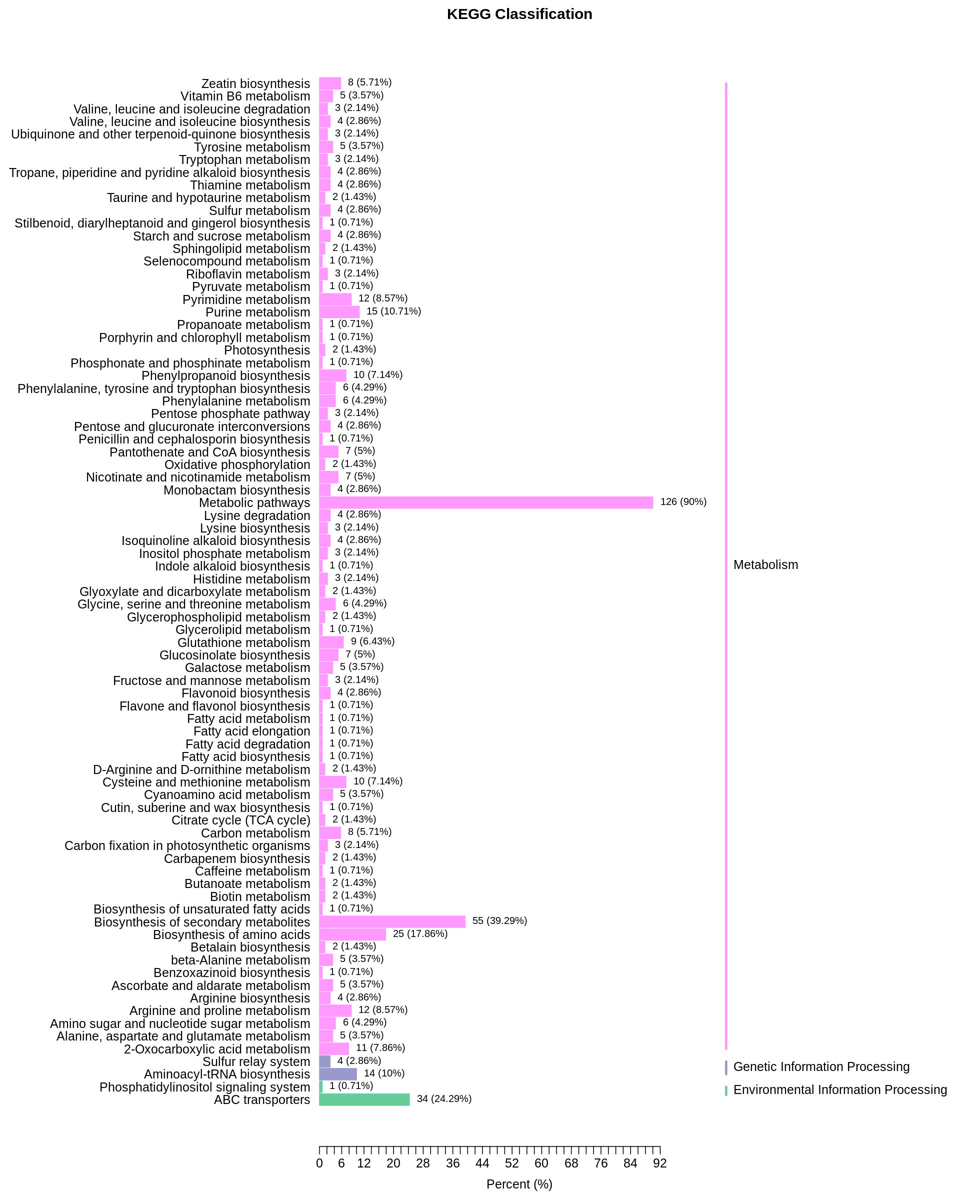


Figure S1 KEGG annotation statistics of metabolites of *Gastrodia elata*.


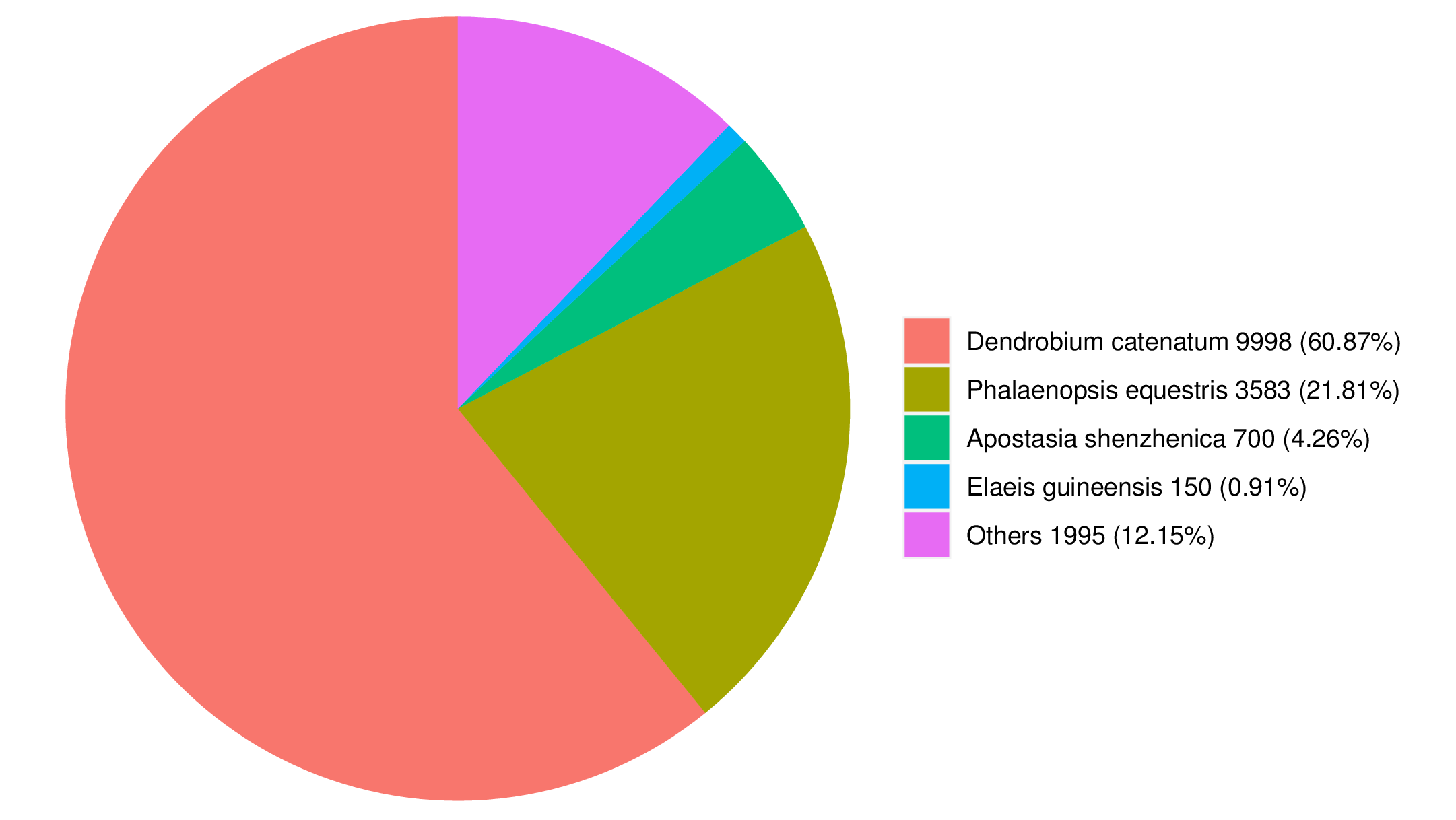


Figure S2 Nr annotation statistics of transcripts of *Gastrodia elata*.


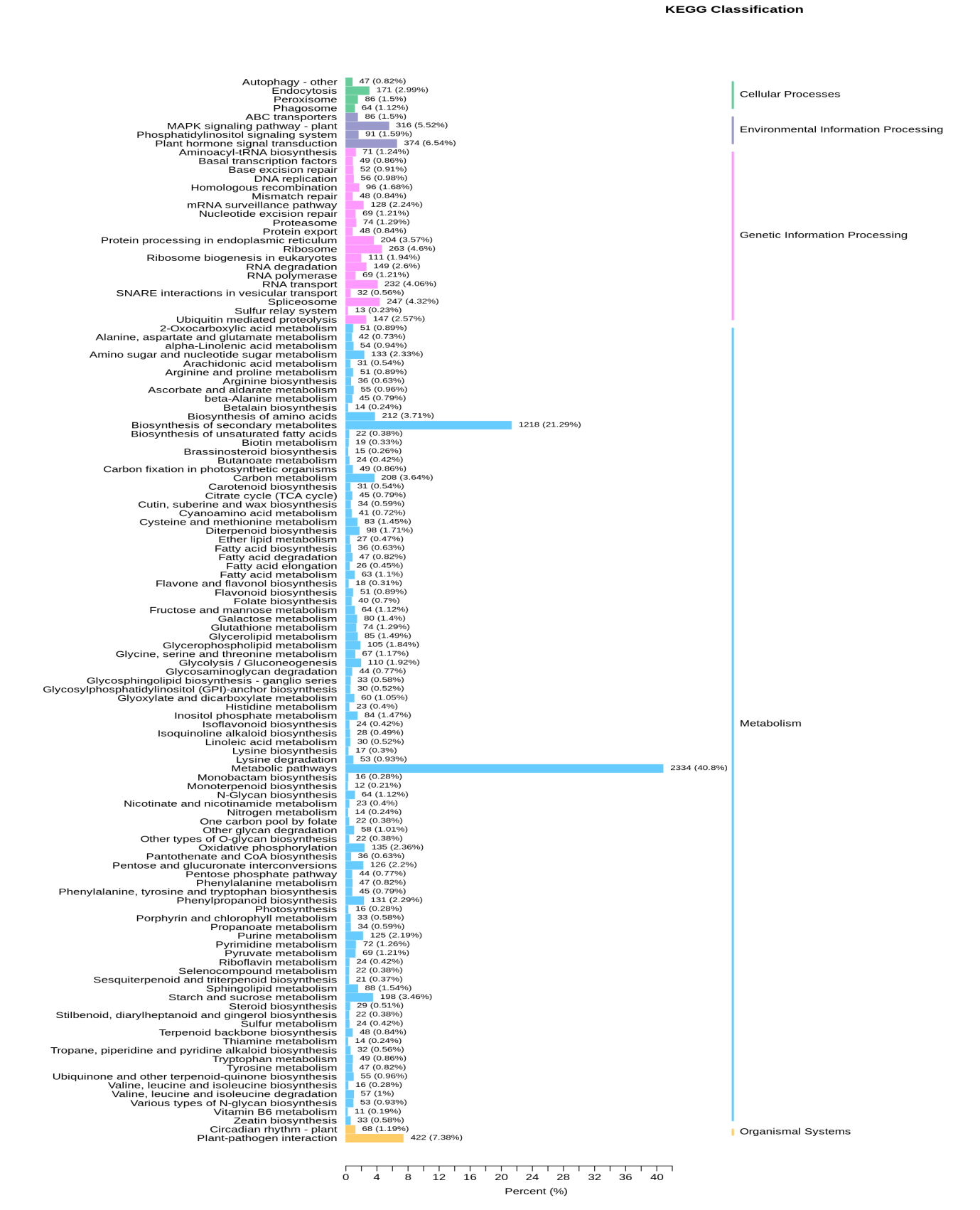


Figure S3 KEGG annotation statistics of transcripts of *Gastrodia elata*.


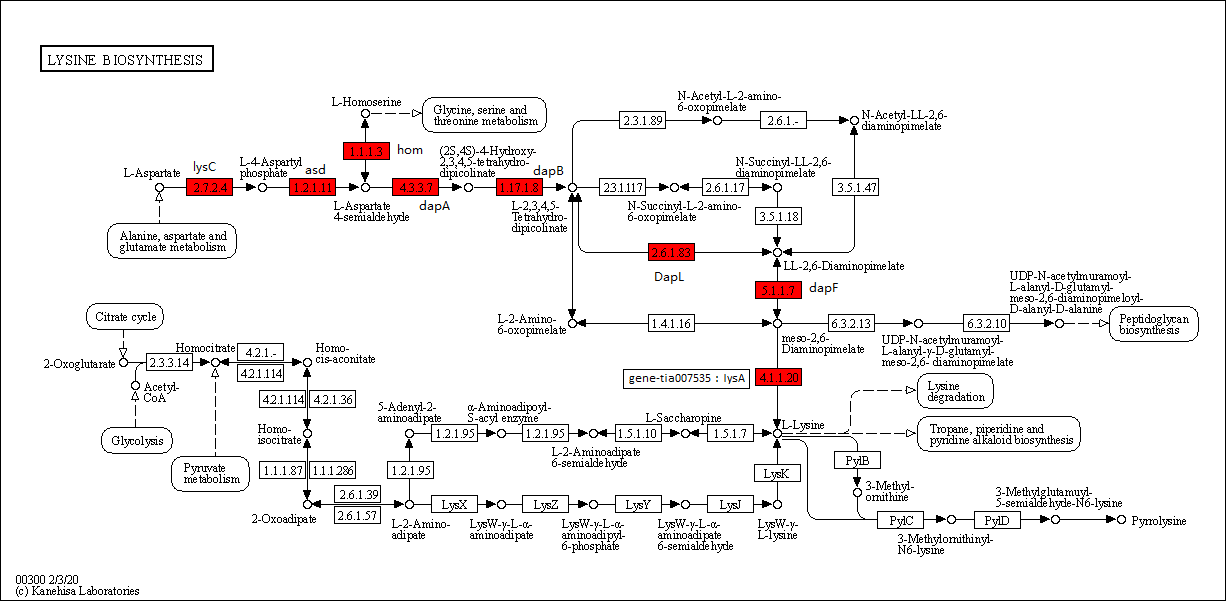


Figure S4 Genes annotated in the lysine biosynthesis pathway

Note: The red box indicates the enzyme annotated by expression genes of tuber tissues of *Gastrodia elata*

*.*


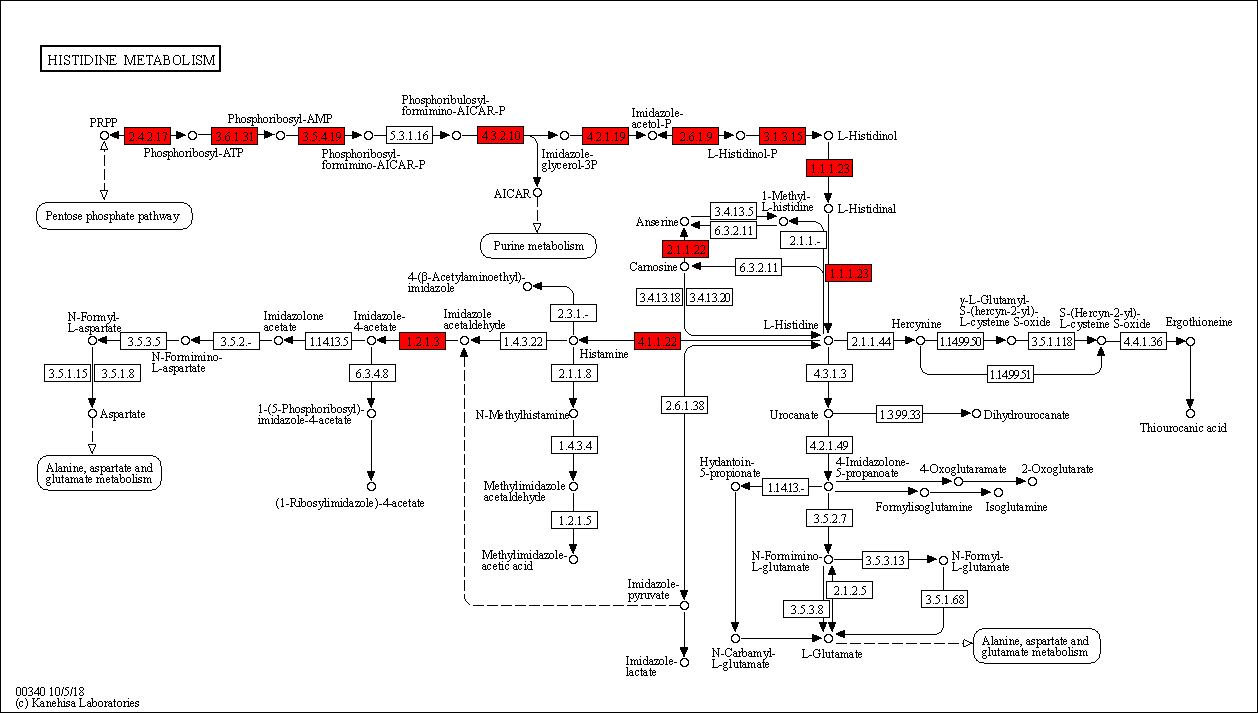


Figure S5 Genes annotated in the histidine metabolism pathways

Note: The red box indicates the enzyme annotated by expression genes of tuber tissues of *Gastrodia elata.*


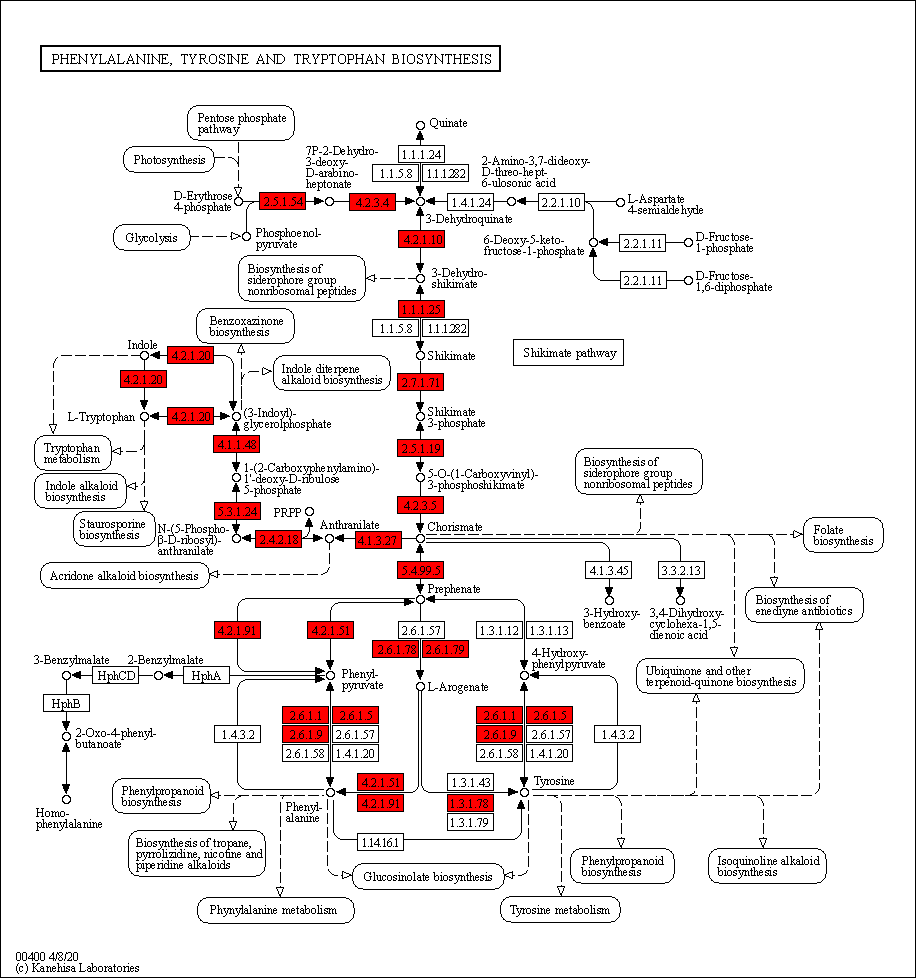


Figure S6 Genes annotated in the phenylalanine, tyrosine and tryptophan biosynthesis pathways

Note: The red box indicates the enzyme annotated by expression genes of tuber tissues of *Gastrodia elata.*


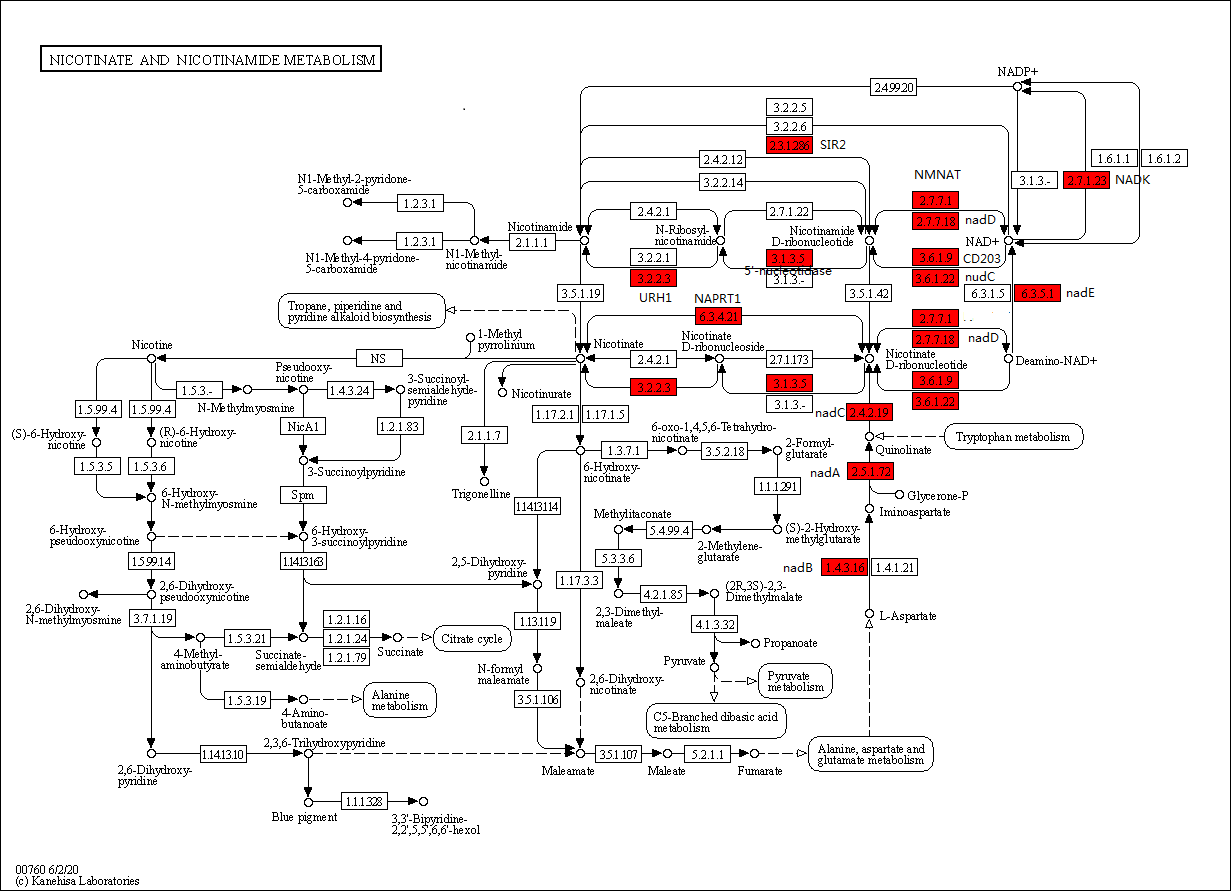


Figure S7 Genes annotated in the nicotinate and nicotinamide metabolism pathways

Note: The red box indicates the enzyme annotated by expression genes of tuber tissues of *Gastrodia elata.*


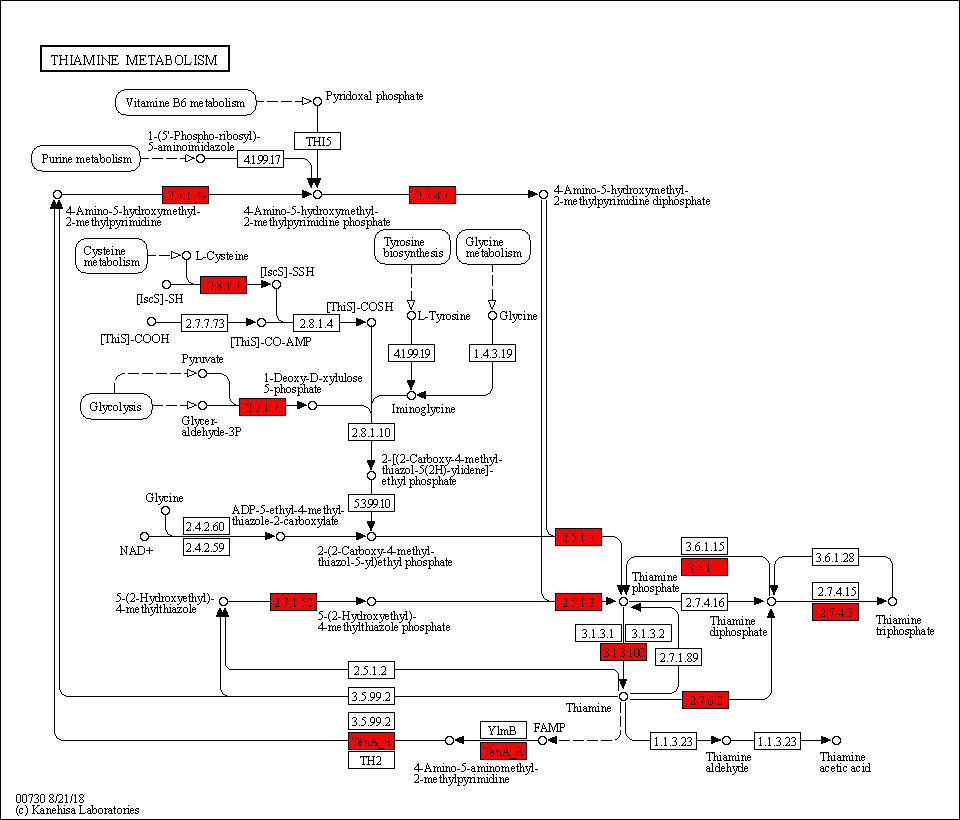


Figure S8 Genes annotated in the thiamine metabolism pathway

Note: The red box indicates the enzyme annotated by expression genes of tuber tissues of *Gastrodia elata.*

*
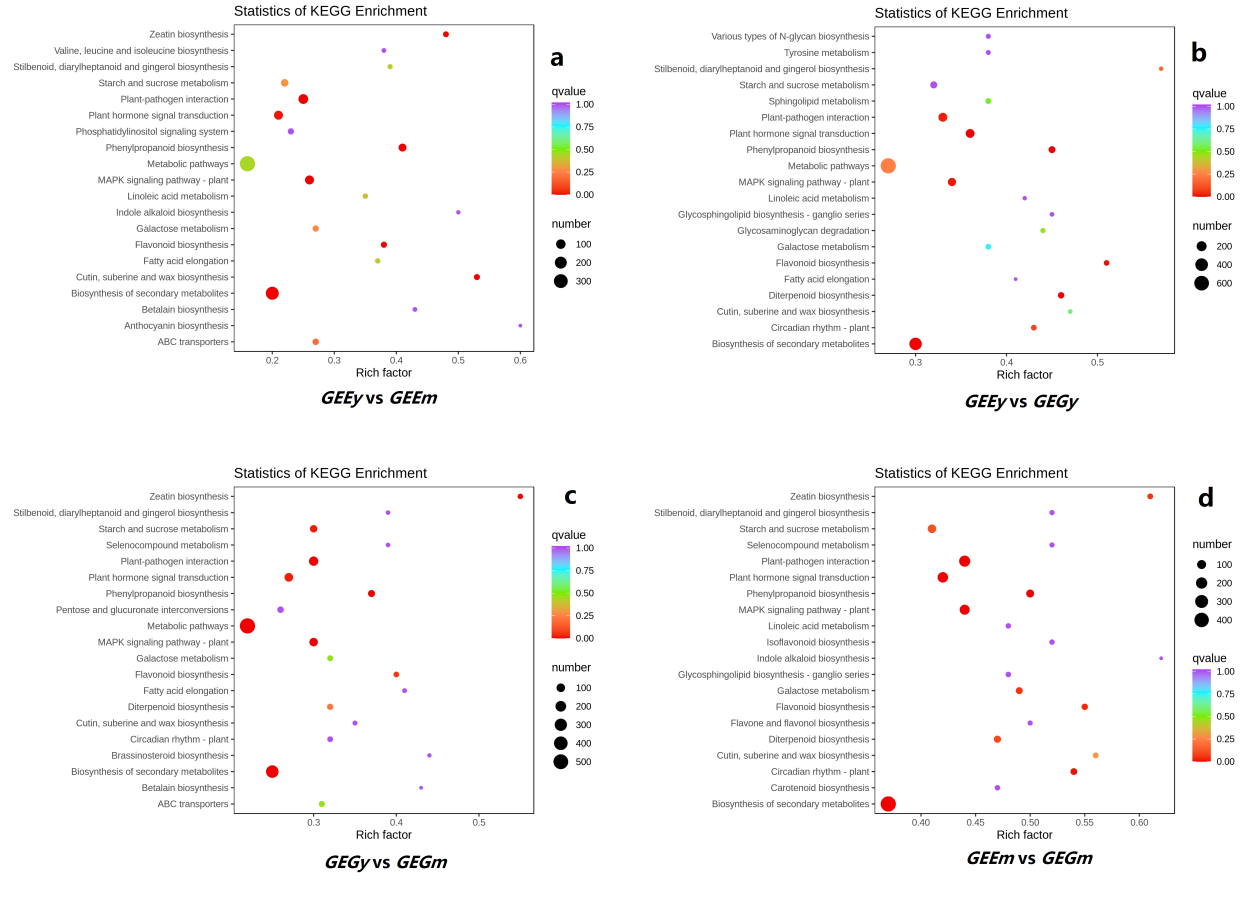
*

Figure S9 KEGG enrichment analysis of DEGs based on (a), *GEEm* vs *GEGm*; (b), *GEEy* vs *GEEm*; (c), *GEGy* vs *GEGm*; and (d), *GEEy* vs *GEGy*

Note: *GEEy* and *GEEm* indicate young and mature tubers of *G. elata.f.elata*, while *GEGy* and *GEGm* represent young and mature tubers of *G. elata.f.glauca*, respectively.
